# Serotonergic 5-HT_7_ Receptors as Modulators of the Nociceptive System

**DOI:** 10.2174/1570159X21666221129101800

**Published:** 2023-05-18

**Authors:** Rita Bardoni

**Affiliations:** 1 Department of Biomedical, Metabolic and Neural Sciences, University of Modena, and Reggio Emilia, Modena, Italy

**Keywords:** Serotonin, pain, 5-HT_7_ receptors, spinal cord, brain, synaptic transmission, endogenous analgesia

## Abstract

The biogenic amine serotonin modulates pain perception by activating several types of serotonergic receptors, including the 5-HT_7_ type. These receptors are widely expressed along the pain axis, both peripherally, on primary nociceptors, and centrally, in the spinal cord and the brain. The role of 5-HT_7_ receptors in modulating pain has been explored *in vivo* in different models of inflammatory and neuropathic pain. While most studies have reported an antinociceptive effect of 5-HT_7_ receptor activation, some authors have suggested a pronociceptive action. Differences in pain models, animal species and gender, receptor types, agonists, and route of administration could explain these discrepancies. In this mini-review, some of the main findings concerning the function of 5-HT_7_ receptors in the pain system have been presented. The expression patterns of the receptors at the different levels of the pain axis, along with the cellular mechanisms involved in their activity, have been described. Alterations in receptor expression and/or function in different pain models and the role of 5-HT_7_ receptors in controlling pain transmission have also been discussed. Finally, some of the future perspectives in this field have been outlined.

## 1 INTRODUCTION

Nociceptive transmission along the pain axis is modulated by the serotonergic system at different levels, from the periphery to the higher centers. Two major nuclei represent the main sources of serotonin (5-HT) in the brain, the nucleus raphe magnus (NRM) and the dorsal raphe nucleus (DRN). The NRM in the rostral ventral medulla projects serotonergic fibers to the spinal cord dorsal horn, especially to the superficial laminae (I-II). DRN sends axonal connections to the large part of the forebrain and several subcortical regions, including the amygdala. While the prevalent function of descending serotonergic pathways is the inhibition of pain in physiological conditions, an imbalance toward facilitation has been detected during pain chronicity, contributing to pain sensitization [1-3]. Maladaptive changes in the activity of serotonergic neurons and modifications of 5-HT receptor expression and function have been found to be involved in the abnormal serotonergic signaling in the different pain states.

Serotonergic receptors (5-HT receptors) belong to seven families (5-HT_1-7_), comprising 14 distinct receptor subtypes (5-HT_1A-F_, 5-HT_2A-C_, 5-HT_3_, 5-HT_4_, 5-HT_5A-B_, 5-HT_6_ and 5-HT_7_). 5-HT_3_ is the only ligand-gated ion channel, while the other receptors belong to the G protein-coupled 7-transmembrane receptor superfamily. The 5-HT_7_ receptor has been identified as a G-protein coupled receptor that stimulates cyclic AMP (cAMP) formation by activating adenylate cyclase through a stimulatory Gs protein. Production of cAMP activates protein kinase A (PKA), inducing the phosphorylation of different target proteins and the activation of multiple signaling cascades, including extracellular signal-regulated kinases (ERK) and protein kinase B (Akt) pathways [4]. The 5-HT_7_ receptor is also coupled to the protein Gα_12_, which in turn activates multiple signaling pathways through the family of small Rho GTPases, Cdc42 and RhoA [5, 6]. In the hippocampus, Gα_12_-mediated signaling undergoes strong developmental regulation, contributing to synaptogenesis and neural network formation [6].

Four mammalian 5-HT_7_ receptor splice variants have been identified that are structurally divergent in the intracellular carboxyl termini: 5-HT_7A,_ 5-HT_7B_, and 5-HT_7C_ in rats and 5-HT_7A,_ 5-HT_7B_, and 5-HT_7D_ in humans. The 5-HT_7_ isoforms differ from each other by the number of N-glycosylation and phosphorylation sites of protein kinases A and C and by the mechanism of internalization [7, 8]. On the other hand, binding affinities to the receptor agonists and functional coupling to adenylate cyclase seem to be similar among the different splice variants [9].

5-HT_7_ receptors have been shown to heterodimerize with 5-HT_1A_ receptors [10]. While 5-HT_7_ receptors stimulate cAMP formation, 5-HT_1A_ activation causes inhibition of adenylate cyclase *via* the Gi protein and subsequent decrease of intracellular cAMP, together with the Gβγ-mediated activation of G-protein-gated inwardly rectifying potassium channels (GIRK). Dimerization of the two receptors decreases the 5-HT_1A_ receptor-mediated activation of Gi protein, reducing the opening of GIRK channels. The coupling of the 5-HT_7_ receptor to the Gs protein does not appear to be significantly affected by heterodimerization [10].

5-HT_7_ receptors are expressed in several areas of the central nervous system (CNS), particularly in the hypothalamus, thalamus, hippocampus, and cerebral cortex [11-13], where they regulate multiple functions, including circadian rhythms, learning and memory, body temperature, and emotional behavior. Targets of 5-HT_7_ receptors on neurons and glial cells are voltage-dependent ion channels, membrane transporters, and synaptic receptors. In general, 5-HT_7_ receptor activation produces neuronal depolarization, an increase in excitability, and an enhancement of neurotransmitter release [14]. These effects are generated by several mechanisms: i) reduction of action potential hyperpolarization through the inhibition of a calcium-dependent potassium current [15]; ii) inhibition of the I_A_ potassium current [16]; iii) enhancement of the hyperpolarization-activated cation current (Ih) through the increase of cAMP [17-19]; and iv) potentiation of voltage-dependent T-type calcium channels [20]. In addition, 5-HT_7_ receptors modulate both excitatory and inhibitory synaptic transmission in several CNS areas by regulating AMPA and NMDA receptor activity [21, 22], GABA release [23-25], and GABA_A_ receptor-mediated currents [26]. In the hippocampus, 5-HT_7_ receptors reverse long-term depression (LTD) mediated by metabotropic glutamate receptors [27, 28], while they induce LTD at the parallel fiber-Purkinje cell synapses in the cerebellum [29].

5-HT_7_ receptors are expressed and functional in many regions involved in the transmission and control of pain. Although their function has not been fully elucidated, several studies suggest an active role in regulating pain sensitivity, especially in chronic conditions. In this mini-review, the most recent findings concerning the role of HT_7_ receptors in acute pain modulation and pain sensitization have been presented.

## 2 5-HT_7_-MEDIATED MODULATION OF PRIMARY NOCICEPTORS AND SECONDARY SENSORY NEURONS

A large number of studies *in vivo* have been carried out to assess the effects of 5-HT_7_ receptors on pain transmission, producing, in some cases, controversial results. Table **1** summarizes the data related to 5-HT_7_-mediated regulation of spinal pain obtained from pharmacological studies or using 5-HT_7_ knockout mice [30, 31]. Some general considerations emerge from these studies: i) 5-HT_7_ receptors do not seem to be importantly involved in acute pain since no significant changes were observed in pain sensitivity in 5-HT_7_ receptor knockout mice or by performing pharmacological studies in acute pain tests [32, 33]; ii) peripheral administration of 5-HT_7_ receptor agonists seems to exert pronociceptive effects in naïve rats [34, 35]; iii) systemic or spinal (intrathecal) administration of 5-HT_7_ agonists produces a prevalent antinociceptive action in animal models of inflammatory and neuropathic pain, while antagonists have pronociceptive effects [32, 36-42]. However, two studies reported a central pronociceptive effect of 5-HT_7_ receptors in the formalin model of inflammatory pain [43] and in rats subjected to spinal nerve injury [44]. Interestingly, spinal 5-HT_7_ receptors have also been involved in the analgesic action of several drugs, including morphine [45], cannabinoids [46], paracetamol [47], and the atypical antidepressant tianeptine [48].

In parallel with the investigation of the role played by 5-HT_7_ receptors *in vivo*, several studies have been performed *in vitro* to characterize the expression and physiology of 5-HT_7_ receptors distributed along the pain axis. In the following paragraphs, some of these findings have been described, obtained from peripheral nociceptors (whose cell bodies are contained in the dorsal root and trigeminal ganglia), and from secondary neurons located in the dorsal horn of the spinal cord and in trigeminal subnucleus caudalis.

### 2.1 Dorsal Root Ganglia

Peripheral 5-HT is a component of the “inflammatory soup” and has been identified as a potent inducer of pain [49, 50] and itch [51, 52]. 5-HT_7_ receptors expressed on dorsal root ganglia (DRG) neurons are believed to contribute to the algogenic and pruritogenic effects of serotonin. The presence of 5-HT_7_ receptors on dorsal root ganglia (DRG) neurons has been demonstrated by several studies: 5-HT_7_ mRNA was reported in both rat and human DRGs, by performing polymerase chain reaction (PCR) [53-56], while 5-HT_7_ immunoreactivity was identified in peptidergic small and medium-sized DRG cell bodies [35, 57]. In rat lumbar DRGs, levels of 5-HT_7_ mRNA increased after the injection into the hind paw of Complete Freund’s Adjuvant (CFA) or bee venom, two models of inflammatory pain [56, 58].

Application of 5-HT or the non-selective 5-HT receptor agonist 5-CT increased the Ih type current in rat medium and large diameter DRG neurons, and the pharmacological profile of this effect was compatible with the activation of 5-HT_7_ receptors [55]. The potentiation was due to a positive shift of the conductance-voltage relationship of the current, leading to the increase of neuronal excitability and action potential firing. Application of the adenylate cyclase activator forskolin produced similar effects, inducing an increase in Ih amplitude and a positive shift of the conductance-voltage relationship. In the presence of forskolin, 5-HT was not able to potentiate Ih, confirming the involvement of a cAMP-dependent signaling pathway in this effect. The 5-HT_7_-mediated potentiation of Ih could affect DRG neurons at different sites: 5-HT_7_ receptors expressed at peripheral terminals could contribute to the algogenic effect of 5-HT released from injured tissues by decreasing nociceptor activation threshold, while receptors located on central terminals could facilitate glutamate and peptide release in the dorsal horn. Furthermore, 5-HT_7_ receptors expressed in the vicinity of DRG somas may be involved in the generation of ectopic discharges under nerve damage and inflammation.

5-HT_7_ receptors present on DRG neurons could also contribute to the sensitization of TRPV1 channels (transient receptor potential channel subfamily V member 1) [59]. In cultured DRG neurons isolated from postnatal rats, 5-HT potentiated capsaicin, protons, and heat-induced increases of intracellular calcium and capsaicin and protons-induced currents. These effects were partially due to 5-HT_7_ receptor activation, being mimicked by the application of the 5-HT_1A_/5-HT_7_ agonist 8-OH-DPAT (which is a preferential 5-HT_1A_ agonist with a moderate affinity for 5-HT_7_ receptors) and significantly suppressed by the 5-HT_7_ antagonist SB-269970. The ability of 5-HT_1A_ and 5-HT_7_ receptors to form heterodimers [10] could influence the results obtained following the application of non-selective 5-HT_7_ agonists. In the same study [59], CFA injection into the hind paw increased 5-HT_7_ mRNA expression in ipsilateral DRGs, in agreement with the results reported by Wu *et al.* [56]. These data are consistent with a pronociceptive role of peripheral 5-HT_7_ receptors; by sensitizing TRPV1 channels, these receptors could contribute to the induction of inflammatory hyperalgesia observed *in vivo*.

A critical role of 5-HT_7_ receptors in 5-HT-dependent acute and chronic itch has also been suggested. As reported by Morita *et al.* [60], the 5-HT-induced itch is decreased in 5-HT_7_ knockout mice, and a correlation has been found between itch behavior and 5-HT_7_ receptor expression in genetically distinct mouse strains. In pruriceptive DRG neurons, the 5-HT_7_ receptor is functionally coupled to the TRPA1 channels (Transient receptor potential channels, subfamily A, member 1); the activation of 5-HT_7_ receptors by the agonist LP-44 promoted the opening of TRPA1 channels *via* cAMP signaling, inducing intracellular calcium elevations. This mechanism could be importantly involved in both acute serotonergic itch and chronic itch linked to elevated levels of 5-HT.

### 2.2 Spinal Cord Dorsal Horn

5-HT_7_ receptors have been identified in the dorsal and ventral horns of the rodent spinal cord, with a prevalence in dorsal horn laminae I and II [35, 38]. Immunoreactivity was less evident in the deep dorsal horn, while moderate staining was observed in motoneurons. Double immunofluorescence labeling of 5-HT_7_ receptors and GABA was revealed in cell bodies of GABAergic interneurons in mice, especially in laminae III-IV [38]. In the same study, 5-HT_7_ receptor expression increased in spinal cord sections of nerve-injured mice. Opposite results were obtained by Amaya Castellanos *et al.* [44], reporting a reduction of receptor protein in both DRG and dorsal horn in spinal nerve-injured rats. An extensive study on 5-HT_7_ receptor expression in the spinal cord dorsal horn has been performed by Doly *et al.* [57], confirming the predominant localization in the superficial laminae. Electron microscopy analysis revealed 5-HT_7_ immunolabelling on cell bodies and dendrites of intrinsic peptidergic neurons, on axon terminals (unmyelinated and thin myelinated peptidergic fibers, belonging to both primary afferents and interneurons), and on astrocytes. Interestingly, labeled dendrites were found postsynaptic to small axon terminals having morphological features of 5-HT fibers, suggesting a synaptic action of 5-HT on 5-HT_7_ receptors. Peptidergic neurons expressing 5-HT_7_ receptors could be represented by both excitatory and inhibitory interneurons, releasing Substance P and enkephalins, respectively.

The presence of 5-HT_7_ receptors on rat spinal GABAergic interneurons has been confirmed by Lin *et al.* [48], showing that the receptors are co-expressed with GAD65 (glutamic acid decarboxylase 65-kilodalton isoform), the enzyme involved in GABA synthesis at axon terminals. Spinal nerve ligation induced mechanical allodynia in neuropathic rats and decreased GAD65 expression and GABA concentration. 5-HT_7_ receptor activation by intrathecal administration of the 5-HT enhancer tianeptine increased GAD65 expression and activity, leading to the enhancement of GABA spinal levels and the attenuation of mechanical allodynia [48].

Data on the cellular mechanisms activated by spinal 5-HT_7_ receptors are still limited. In rat deep dorsal horn neurons, the 5-HT_1A_/_7_ receptor agonists 8-OH-DPAT and 5-CT facilitated glutamatergic synaptic responses evoked by dorsal root stimulation in slices [61]. The effect was likely mediated by the 5-HT_7_ receptor since it was reversed by subsequent application of the 5-HT_7_ receptor antagonist clozapine, while it was not affected by the 5-HT_1A_ antagonist NAN-190.

Bannister *et al.* [62] have described the involvement of spinal 5-HT_7_ receptors in the mechanism of diffuse noxious inhibitory control (DNIC), a form of descending endogenous analgesia, able to reduce the activity of wide dynamic range (WDR) neurons. DNIC is normally present in naïve animals but absent in spinal nerve-ligated rats, a model of neuropathic pain. Spinal application of selective serotonin reuptake inhibitors (SSRIs) induced DNIC in neuropathic rats, and this effect was abolished upon application of SSRIs plus the 5-HT_7_ antagonist SB-269970. These results provide evidence that an excess of spinal 5-HT (induced by SSRI administration) can activate 5-HT_7_ receptors in the deep dorsal horn, restoring DNIC on WDR neurons.

As shown in a recent study, the application of the 5-HT_7_ agonist LP-211 to mouse spinal cord slices modulates both glutamatergic and GABA/glycinergic transmission in lamina II [63]. These effects were blocked by SB-269970, confirming the involvement of 5-HT_7_ receptors. Analysis of LP-211 action on both spontaneous and stimulation-evoked synaptic transmission revealed a predominant facilitatory effect on inhibitory transmission, together with an increase in the excitability of tonic firing cells (likely representing inhibitory interneurons). The increase in GABA release could exert inhibitory effects both at post- and presynaptic sites in the dorsal horn by decreasing the excitability of postsynaptic neurons and inhibiting glutamate release from primary afferents [64]. The involvement of GABAergic transmission in spinal 5-HT_7_ receptor modulation has also been proposed by Viguier *et al.* [39], showing a reduction in the analgesic effects of 5-HT_7_ agonists in neuropathic rats by blocking spinal GABA_A_ receptors with bicuculline.

These data support a predominant analgesic action of 5-HT_7_ receptors in the spinal cord; it is possible that the antinociception mediated by spinal 5-HT_7_ receptors overcomes the pronociceptive action exerted at the periphery, leading to the overall analgesic effect found after systemic administration of agonists in animal models of pain sensitization (Table **1**).

### 2.3 Trigeminal Subnucleus Caudalis

Expression of 5-HT_7_ receptors has been detected in trigeminal ganglia (TG) neurons in both humans and rats [65, 66]. In rats, most TG neurons expressing 5-HT_7_ receptors are peptidergic, containing CGRP (Calcitonin Gene Related Peptide) or substance P [66]. Nociceptive TG neurons innervate the orofacial region project centrally to the trigeminal subnucleus caudalis (Vc or medullary dorsal horn). Using the perforated patch-clamp technique, Yang *et al.* [67] reported that 8-OH-DPAT induced the depolarization of a subpopulation of neurons in mouse Vc *Substantia gelatinosa* (SG). The effect was blocked by the 5-HT_7_ antagonist SB-269970 and was not affected by the 5-HT_1A_ antagonist WAY 100-635. The depolarization was due to the direct activation of 5-HT_7_ receptors on SG cells since it persisted in the presence of tetrodotoxin, AMPA, NMDA, GABA_A_, and glycine receptor blockers. Consistently, single-cell RT-PCR revealed the presence of 5-HT_7_ mRNA in about 25% of Vc neurons. Furthermore, larger depolarizing responses to 8-OH-DPAT were observed in postnatal P5-21 mice compared to P22-84 mice, suggesting a decrease in the 5-HT_7_ receptor expression during development. An age-dependent expression of 5-HT_7_ receptors had also been observed in other CNS areas, such as rat prefrontal cortex [68] and mouse hippocampus [10], consistently with a role of these receptors in neurogenesis.

The presence of 5-HT_7_ receptors in TG and Vc neurons and their ability to excite Vc neurons are compatible with the role of these receptors in regulating oro-facial pain. In a model of trigeminal pain in mice, the formalin injection into the upper lip and systemic administration of brain-penetrant 5-HT_7_ agonist LP-211 reduced face rubbing time in both phases I and II [69], where phase I corresponded to acute pain and phase II depended on the inflammatory reaction in the peripheral tissues and on functional changes in the brain stem. Similar results were obtained following systemic administration of the 5-HT_7_ agonist LP-44, although the rapid catabolism of this compound may complicate the interpretation of experiments performed *in vivo*. 5-HT_7_ receptors located in the Vc SG could contribute to the antinociceptive action of LP-44 and LP-211, especially during phase II. Analogously to the spinal cord, 5-HT_7_ receptors could exert a depolarizing action prevalently on inhibitory interneurons, determining a net antinociceptive effect.

A role of 5-HT_7_ receptors in the pathogenesis of migraine has also been proposed; increased levels of 5-HT released from perivascular serotonergic fibers could target 5-HT_7_ receptors expressed on smooth muscle cells of large cranial vessels, inducing vasodilation, activation of trigeminal sensory nerves, and release of pro-inflammatory peptides (CGRP, Substance P) [70]. Consistently, the 5-HT_7_ receptor antagonist SB-269970 reduced neurogenic dural vasodilation [71] and CGRP release [72] in rats. 5-HT_7_ receptors expressed on astroglial “feet” [57] could also be actively involved in the pathogenesis of migraine by regulating cranial vessel function.

## 3 5-HT_7_ RECEPTORS AS PAIN MODULATORS IN HIGHER BRAIN CENTERS

### 3.1 Dorsal Raphe Nucleus and Periaqueductal Grey Area

The dorsal raphe nucleus (DRN) is the main source of 5-HT projections to the forebrain, which regulate the activity of neuronal circuits involved in numerous functions, such as emotional states, sleep, and motivation. The DRN has also been shown to have an inflammation-specific antinociceptive function in rats subjected to the formalin test [73]. 5-HT_7_ receptors are expressed and functional in DRN; electrophysiological recordings from presumed projection neurons in rat DRN slices revealed that the receptors are involved in the modulation of GABA release [74]. The activation of 5-HT_7_ receptors by 5-CT (in the presence of the 5-HT_1A_ antagonist WAY-100635) caused the increase of spontaneous GABAergic synaptic currents and the subsequent hyperpolarization of the recorded cells. 5-HT_7_ receptors in DRN slices were tonically active since the application of the antagonist SB-269970 resulted in cell depolarization and an increase of action potential firing. Further experiments will be necessary to assess the role of this mechanism in the antinociceptive action of DRN.

Midbrain periaqueductal grey area (PAG) plays a critical role in endogenous analgesia by activating descending serotonergic and noradrenergic pathways projecting to the dorsal horn. 5-HT_7_ receptors expressed in PAG contribute to the antinociceptive function of this area; injection of the 5-HT_7_ agonist AS-19 into rat ventrolateral PAG exerted an anti-hyperalgesic effect in the chronic constriction injury model of neuropathic pain that was attenuated by SB-269970 [75]. Since the analgesic action of AS-19 was partially reduced by an antagonist of the purinergic P_2X3_ receptor, a possible potentiating effect of 5-HT_7_ receptors on P_2X_ receptors has been proposed.

### 3.2 Medial Thalamus

The role of 5-HT_7_ receptors in pain modulation has also been investigated in the medial thalamic parafascicular nucleus (nPf), which is implicated in the processing of the affective component of pain. This area receives spinothalamic nociceptive afferents and projects to the anterior cingulate cortex. In addition, the nPf receives serotonergic projections from the DRN and ventrolateral PAG and expresses 5-HT_1A_ and 5-HT_7_ receptors [76, 77]. As shown by Harte *et al.* [78], 8-OH-DPAT administration into the rat nPf increased vocalization thresholds elicited by tail shocks. The effect was reversed by both 5-HT_1A_ and 5-HT_7_ antagonists, suggesting the involvement of both receptors in the antinociceptive effect of 8-OH-DPAT. Since the activation of thalamic 5-HT_7_ receptors has been reported to facilitate neuronal excitation [15, 17], part of the antinociceptive effect of 8-OH-DPAT could be due to the activation of inhibitory thalamic interneurons, such as the enkephalinergic neurons [78].

### 3.3 Ventrolateral Orbital and Anterior Cingulate Cortex

The ventrolateral orbital cortex (VLO) is part of a descending endogenous analgesic system that reaches the spinal cord *via* the PAG. Xu *et al.* [79] have shown that microinjection of 5-HT into the VLO depresses mechanical allodynia in the rat spared injury model of neuropathic pain. Injection of the antagonist SB-269970 into VLO attenuated the 5-HT-induced inhibition of allodynia, demonstrating a contribution of 5-HT_7_ receptors to the 5-HT-mediated antinociceptive effect.

The anterior cingulate cortex (ACC) is one of the key brain regions for the processing of pain, involved in encoding the emotional and aversive aspects of nociception. In chronic pain states, ACC undergoes maladaptive plasticity changes, leading to hyperactivity, an increase in excitability, and synaptic reorganization [80-82]. As reported by Santello *et al.* [83], the hyperpolarization-activated cyclic nucleotide-gated (HCN) channels, responsible for the Ih current and expressed on the dendrites of ACC pyramidal neurons, underwent functional downregulation in the mouse chronic constriction injury model. This, in turn, increased the summation of excitatory postsynaptic potentials (EPSP) in the dendrites, generating a state of hyperexcitability. Activation of 5-HT_7_ receptors by LP-211 in ACC slices potentiated HCN channels by elevating cAMP levels, thus reducing EPSP summation and neuronal excitability. This mechanism could be involved in the analgesia produced by systemic injection of LP-211 after nerve injury, partially inhibited by the injection of SB-269970 into ACC [84]. Interestingly, the 5-HT_7_ antagonist was ineffective when injected into the somatosensory cortex SI, suggesting a more critical role of cortical 5-HT_7_ receptors in modulating the affective components of pain compared to sensory discrimination.

## CONCLUSION AND FUTURE PERSPECTIVE

**Fig. (1):**
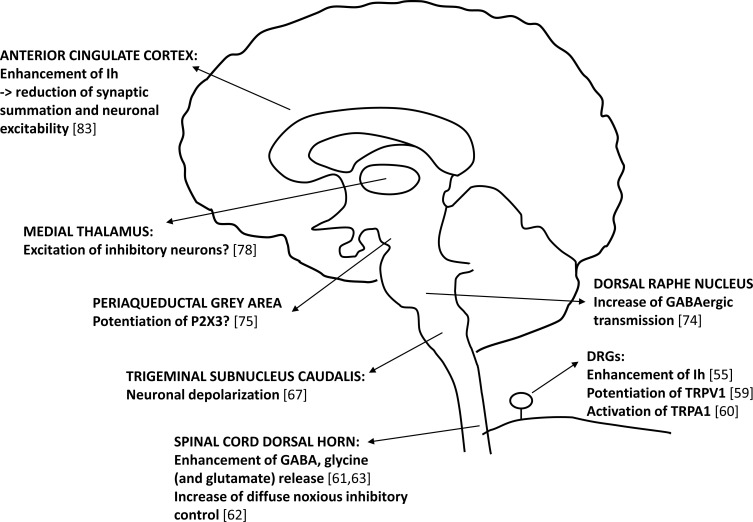
Schematic representation of the cellular mechanisms activated by 5-HT_7_ receptors along the pain axis. Ion channels and synaptic neurotransmitters/receptors modulated by 5-HT_7_ receptors are reported for each area. Question marks are referred to mechanisms not yet demonstrated. References are indicated inside square brackets.

The studies described in this mini-review strongly suggest an important involvement of 5-HT_7_ receptors in pain modulation, both at the periphery and in the CNS (Fig. **1**). The emerging view is that receptors expressed peripherally on primary nociceptors exert a prevalent pronociceptive action, while 5-HT_7_ receptors located in the CNS play a predominant antinociceptive role.

However, many aspects are still not clear, and further investigation is required. For example, the antinociceptive role of 5-HT_7_ receptors in the CNS has been questioned by some studies reporting opposite results. These discrepancies could derive from the heterogeneity of species, the pain models, and the use of non-selective 5-HT_7_ agonists. The recruitment of different 5-HT_7_ receptor isoforms and/or of 5-HT_1A_/5-HT_7_ heterodimers could also contribute to a variety of results. More standardized experimental conditions and the use of more selective agonists, together with the employment of animals where 5-HT_7_ receptors are silenced or not expressed, would be helpful to clarify these issues.

In addition, most behavioral studies testing the effects of 5-HT_7_ receptor agonists or antagonists were obtained from male rodents (Table **1**). Interestingly, a pronociceptive action of 5-HT_7_ receptors in the CNS was mainly described by studies performed on female rats [43, 44], while tests carried out on males showed a prevalent antinociceptive effect. A sex-dependent activity of 5-HT_7_ receptors would be consistent with many studies reporting the influence of gender on the serotonergic system. Indeed, sex-related differences in 5-HT content, serotonergic turnover, and 5-HT receptor expression and function have been detected in several CNS areas [85, 86].

The interpretation of the behavioral studies has been found difficult due to the lack of understanding of the neural circuits and cellular mechanisms involved in 5-HT_7_-mediated pain modulation. More recent technical approaches, such as the fluorescent labeling of specific neuronal populations and/or the chemo- and optogenetic activation of identified neuronal classes, will be critical to elucidate the role of 5-HT_7_ receptors in both acute and persistent pain. However, the understanding of the functional alterations occurring in the serotonergic system in chronic pain states is very complex; several factors, including the levels of 5-HT released, the targeted neuronal types, and the classes of 5-HT receptors activated, could alter the balance between 5-HT inhibitory and excitatory effects and drive the nociceptive system toward a state of hyperexcitability.

Finally, the antinociceptive action of many 5-HT_7_ receptor agonists would suggest a therapeutic application of these compounds in the treatment of chronic pain. However, the paucity of highly selective, brain-penetrant, and metabolically stable agonists, together with the involvement of the receptors in multiple physiological functions and diseases and their functional interactions with other 5-HT receptors, has made the development of analgesic compounds targeting 5-HT_7_ receptors particularly challenging. A new, orally bioavailable compound (AGH-192), endowed with high metabolic stability and selectivity for 5-HT_7_ receptors, has been recently described as a potential analgesic, effective in the treatment of neuropathic pain [42]. Furthermore, a biased ligand of 5-HT_7_ receptors, serodolin, has been recently tested for its therapeutic potential [87]. This compound acts as a potent inverse agonist for Gs signaling while inducing an agonistic response on the ERK pathway. Serodolin-induced ERK activation employs a β-arrestin signaling mechanism that is independent of Gs and requires c-SRC activation. Systemic administration of serodolin decreased hyperalgesia and pain response in several pain models in mice, while no effects of the compound were observed in the presence of SB-269970 or in 5-HT_7_ knockout mice. These findings demonstrate the involvement of β-arrestin signaling in 5-HT_7_-mediated analgesia, exhibiting a potential for the therapeutic application of 5-HT_7_ β-arrestin-biased ligands.

## Figures and Tables

**Table 1:** 5-HT_7_-mediated modulatory effects on spinal pain.

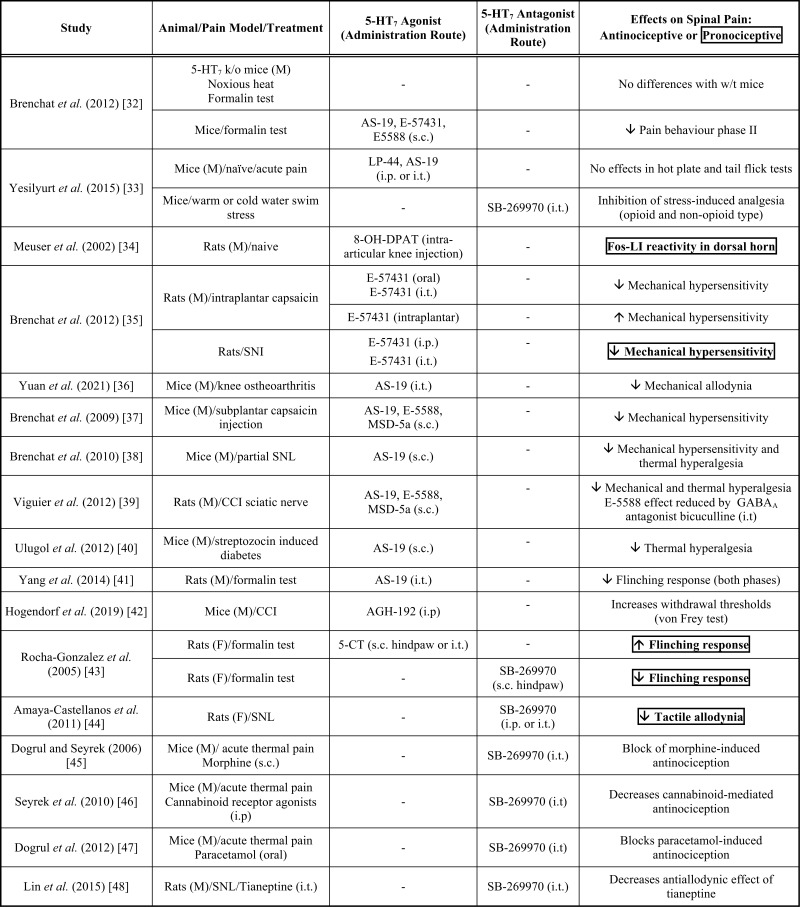

## LIST OF ABBREVIATIONS

AGH-192: 3-(1-ethyl-1H-imidazol-5-yl)-5-iodo-4-fluoro-1H-indole
AS-19: (2*S*)-(+)-5-(1,3,5-Trimethylpyrazol-4-yl)-2-(dimethylamino)tetralin
5-CT: -Carboxamidotryptamine
E-55888: N,N-Dimethyl-2-[3-(1,3,5-trimethylpyra-zol-4-yl)phenyl]ethanamine
E-57431: (2‐(2‐(dimethylamino)ethyl)‐4‐(1,3,5‐trimethyl‐1H‐pyrazol‐4‐yl)phenol hydrochloride)
MSD-5a: Dimethyl-[2-(6-phenyl-pyridin-2-ylsul-fanyl)-ethyl]-amine hydrochloride
NAN-190: 1-(2-Methoxyphenyl)-4-(4-phthalimido-butyl)piperazine
8-OH-DPAT: 8-hydroxy-2-(di-n-propylamino) tetralin
SB-269970: (R)-3-(2-(2-(4-methylpiperidin-1-yl)ethyl)pyrrolidine-1-sulfonyl) phenol
WAY-100635: N-[2-[4-(2-Methoxyphenyl)-1-pipera-zinyl]ethyl]-N-2-pyridinylcyclohexane-carboxamide
